# Knockdown of CNN3 Impairs Myoblast Proliferation, Differentiation, and Protein Synthesis via the mTOR Pathway

**DOI:** 10.3389/fphys.2021.659272

**Published:** 2021-07-08

**Authors:** Yanling She, Cheng Li, Ting Jiang, Si Lei, Shanyao Zhou, Huacai Shi, Rui Chen

**Affiliations:** ^1^Guangdong Traditional Medical and Sports Injury Rehabilitation Research Institute, Guangdong Second Provincial General Hospital, Guangzhou, China; ^2^Department of Radiology, The Third Affiliated Hospital, Sun Yat-sen University, Guangzhou, China

**Keywords:** CNN3, C2C12 myoblasts, proliferation, differentiation, protein synthesis, mTOR pathway

## Abstract

**Background:**

Myogenesis is a complex process that requires optimal outside–in substrate–cell signaling. Calponin 3 (CNN3) plays an important role in regulating myogenic differentiation and muscle regeneration; however, the precise function of CNN3 in myogenesis regulation remains poorly understood. Here, we investigated the role of CNN3 in a knockdown model in the mouse muscle cell line C2C12.

**Methods:**

Myoblast proliferation, migration, differentiation, fusion, and protein synthesis were examined in CNN3 knockdown C2C12 mouse muscle cells. Involvement of the mTOR pathway in CNN3 signaling was explored by treating cells with the mTOR activator MHY1485. The regulatory mechanisms of CNN3 in myogenesis were further examined by RNA sequencing and subsequent gene ontology (GO), Kyoto Encyclopedia of Genes and Genomes (KEGG) and gene set enrichment analysis (GSEA).

**Results:**

During proliferation, CNN3 knockdown caused a decrease in cell proliferation and migration. During differentiation, CNN3 knockdown inhibited myogenic differentiation, fusion, and protein synthesis in C2C12 cells via the AKT/mTOR and AMPK/mTOR pathways; this effect was reversed by MHY1485 treatment. Finally, KEGG and GSEA indicated that the NOD-like receptor signaling pathway is affected in CNN3 knockdown cell lines.

**Conclusion:**

CNN3 may promote C2C12 cell growth by regulating AKT/mTOR and AMPK/mTOR signaling. The KEGG and GSEA indicated that inhibiting CNN3 may activate several pathways, including the NOD-like receptor pathway and pathways involved in necroptosis, apoptosis, and inflammation.

## Introduction

Skeletal muscle occupies∼40% of the mammalian body and is essential for maintaining posture, locomotion, blood circulation, breathing, and metabolism (Zurlo et al., 1990; Simionescu-Bankston and Kumar, 2016). Skeletal muscle loss occurs at a rate of 1–5% annually from the age of 30 years; this is related to worse outcomes after falls in older age groups and is a compelling reason for physical therapy interventions (Avers and Brown, 2009). Therefore, it is vital to prevent skeletal muscle loss.

Calponin (CNN), which was first identified in chicken gizzard smooth muscle, induces actin polymerization (Takahashi et al., 1986; Rozenblum and Gimona, 2008). There are three genetic isoforms of CNN: h1 or basic CNN (CNN1), h2 or neutral CNN (CNN2), and h3 or acidic CNN (CNN3) (Hirata et al., 2016; Liu and Jin, 2016), which have largely conserved structures (Liu and Jin, 2016; Feng et al., 2019). It has been showed that both CNN2 and CNN3 were highly expressed in C2C12 cells (Shibukawa et al., 2010; Jiang et al., 2014). CNN3 binds to F-actin and is essential for cytoskeletal rearrangement and wound healing (Daimon et al., 2013; Ciuba et al., 2018). CNN3 is highly expressed in the brain (Junghans and Herzog, 2018), cancers (Hong et al., 2019; Nair et al., 2019; Xia et al., 2020), smooth muscle (Ma et al., 2020), and human placenta cytotrophoblasts (Shibukawa et al., 2010; Appel et al., 2014). High-throughput transcriptomic analysis in patients with hematological malignancies revealed that CNN3 expression can be used as a marker of systemic inflammatory response syndrome or sepsis (Labiad et al., 2018). Loss of CNN3 in embryonic neuronal stem cells increases the number of newly formed neurospheres and affects neurosphere size by activating Wnt signaling pathways (Junghans and Herzog, 2018). CNN3 is also a negative regulator of trophoblast fusion. Furthermore, depletion of CNN3 promoted actin cytoskeletal rearrangement and syncytium formation in human choriocarcinoma cell line (Shibukawa et al., 2010). CNN3 plays an important role in stress fiber formation in primary fibroblasts, as CNN3 knockdown led to a decrease in cell motility and contractility (Daimon et al., 2013). In lens epithelial cells and explants, reduced CNN3 expression augmented contractility, plasticity, fibrogenic activity, and mechanosensitive Yap/Taz transcriptional activation (Maddala et al., 2020). In nude rats transplanted with human skeletal myoblasts in infarcted myocardium, the expression of CNN3 was upregulated 1 month after transplant (Perez-Ilzarbe et al., 2008). CNN3 is also associated with skeletal muscle growth (Shibukawa et al., 2013; Tang et al., 2014); however, how CNN3 regulates myogenesis remains largely unknown.

Hyperactivation of mammalian target of rapamycin (mTOR) signaling increases cell and tissue growth (Matsumoto et al., 2017). mTOR is activated by insulin, nutrients, growth factors, stress, and cellular energy (Choi et al., 2016). AMP-activated protein kinase (AMPK) is an energy sensor essential for cellular growth and energy homeostasis (Joshi-Barr et al., 2014). AMPK regulates mTOR by phosphorylating it at Thr2446, which prevents AKT-mediated phosphorylation of mTOR at Ser2448 (Kj bsted et al., 2018). The AKT/mTOR pathway is a crucial regulator of skeletal muscle and is downregulated during muscle atrophy and upregulated during hypertrophy *in vivo* (Scrimgeour et al., 2001). In addition, we recently found that mTOR is involved in the regulation of autophagy and muscle protein degradation under hypoxic conditions (Chen et al., 2017). It is essential to investigate the effects of mTOR on muscle cells to gain a better understanding of myogenesis.

In this study, we investigated the function and mechanisms of CNN3 during myogenesis. This work has revealed novel mechanisms of CNN3 and suggests that CNN3 regulates the mTOR pathway during muscle development.

## Materials and Methods

### Animals

Male C57BL/6 mice (5 weeks old) were obtained from the Animal Laboratory of Sun Yat-sen University. All mice were acclimated to the conditions for 1 week before experimentation. The mice were maintained under standard conditions of 22 ± 2°C, 50–60% relative humidity, and alternate dark–light cycles. Food and water were provided *ad libitum*. Mice were injected with 100 μL of 10 μM cardiotoxin (CTX; Zhongxin Dongtai Nano-Gene Biotechnology Co., Ltd., China) into the hindlimb muscles (Singh et al., 2014). The tibialis anterior muscles were harvested and processed for analysis at 0, 1, 3, 5, 7, and 14 days after injection. All animal procedures were according to the Suggestions for the Care and Use of Laboratory Animals provided by the Ministry of Science and Technology of the People’s Republic of China. This study was approved by the Ethics Committee of Guangdong Second Provincial General Hospital (no: 20200622-GZSKJ-59).

### C2C12 Cell Culture

The C2C12 mouse myoblast line (Stem Cell Bank, Chinese Academy of Sciences, Shanghai, China) was cultured in Dulbecco’s modified Eagle medium (DMEM), high glucose (Gibco; Thermo Fisher Scientific, Inc., Waltham, MA, United States) supplemented with 10% fetal bovine serum (Gibco; Thermo Fisher Scientific, Inc.,), 100 U/mL penicillin, and 100 μg/mL streptomycin in 5% CO_2_ at 37°C. When the cells reached 80–90% confluence, differentiation was induced by incubation in DMEM containing 2% horse serum (Gibco; Thermo Fisher Scientific, Inc.,).

### *In vitro* siRNA Transfection

C2C12 cells (5 × 10^4^) were seeded in six-well plates and incubated in 5% CO_2_ at 37°C for 8 h until the cell density attained was 30–40%. A solution of 60 nM siRNA (Shanghai GenePharma Co., Ltd., Shanghai, China) and 4.5 μL Lipofectamine RNAiMAX Reagent (Thermo Fisher Scientific, Inc.) was incubated at room temperature for 15 min and then added dropwise onto cells. For cell differentiation, the transfection was repeated at 72 h after the first transfection. The cells were harvested at 48 and 144 h for proliferation and differentiation analyses, respectively. The siRNA sequences are shown in Supplementary Table 1 and the timeline of the experiments is showed in Supplementary Figure 1.

### qRT-PCR

Total RNA was extracted from cells using TRIzol reagent (Takara Biotechnology Co., Ltd., Otsu, Japan) according to the manufacturer’s protocol. The RNA (500 ng) was reverse-transcribed into cDNA using PrimeScript^TM^ RT Master Mix (Takara Biotechnology Co., Ltd.). The resulting cDNA was subjected to PCR using SYBR^®^ Green Mix (Takara Biotechnology Co., Ltd.) to quantify mRNA expression levels. The PCR parameters were as follows: 30 s Taq activation at 95°C, followed by 40 cycles of denaturation at 95°C for 5 s, annealing at 60°C for 30 s, and extension at 72°C for 1 min. The analyses were performed using the StepOnePlus^TM^ Real-Time PCR System (Applied Biosystems, Waltham, CA, United States), and gene expression is expressed relative to 18S RNA. The primer sequences used for PCR are shown in Supplementary Table 2.

### Western Blot Analysis

To extract total proteins, C2C12 cells were lysed using RIPA buffer containing a protease inhibitor (Beyotime Institute of Biotechnology, Jiangsu, China) and phenylmethylsulfonyl fluoride. Equal amounts of protein (20 μg) were separated by 10–12% sodium dodecyl sulfate polyacrylamide gel electrophoresis and transferred onto polyvinylidene fluoride membranes. The membranes were blocked with 5% non-fat milk and incubated with a primary antibody targeting CNN3 (1:10,000; ab151427, Abcam, Cambridge, United Kingdom), CNN2 (1:500; DF3870, Affinity, Cincinnati, OH, United States), MEF2A (1:10,000; ab109420, Abcam), myogenin (Myog; 1:500; MAB3876, EMD Millipore, Billerica, MA, United States), puromycin (1:25,000; MABE343, Millipore), myosin heavy chain (MyHC; 1:1,000; MAB4470, R&D Systems, Inc., Minneapolis, United States), CDK2 (1:1,500; A18000, ABclonal Biotech, Co., Ltd., Woburn, MA, United States), CDK4 (1:1,500; A11136, ABclonal Biotech), CDK6 (1:1,000, A1545, ABclonal Biotech), p-AMPK (1:1,000; 2535, Cell Signaling Technology, Inc., Danvers, MA, United States), AMPK (1:1,000; 5832; CST), p-AKT (1:5,000; ab81283, Abcam), AKT (1:1,000; 4691, CST), p-mTOR (1:1,000; 5536, CST), or mTOR (1:1,000; 2983, CST) overnight at 4°C. The membranes were then incubated with goat anti-mouse (1:10,000; AS003, ABclonal Biotech) or anti-rabbit secondary (1:10,000; AS014, ABclonal Biotech) antibody for 1 h at room temperature. Band intensity was determined using a chemiluminescent imaging system (Tanon Sciences and Technology Co., Ltd., Shanghai, China). Tubulin (1:5,000; AC021, ABclonal Biotech) was measured as an internal control for protein quantification. Band intensity was quantified using Image J software (v1.4.3.67, National Institutes of Health, Bethesda, MD, United States).

### Cell Viability Assays

Cell viability was assessed using Cell Counting Kit 8 (CCK8) assays (Beyotime Institute of Biotechnology). Briefly, C2C12 cells were seeded onto 96-well plates at 0.2 × 10^4^/well. Following siRNA transfection for 24, 48, or 72 h, the cells were treated with CCK8 solution. After 1 h incubation, the absorbance at 450 nm was measured using a microplate reader (BioTek Instruments, Inc., Winooski, Vermont, United States). Data are presented as the percentage of the control.

### EdU Staining

Cell proliferation was detected using an EdU kit (KeyGEN Biotech Co., Ltd., Jiangsu, China) according to the manufacturers’ instructions after transfecting with siRNA for 48 h. The cells were cultured in 10 μM EdU working solution for 2 h prior to the end of culture. The cells were fixed in 4% polyformaldehyde in phosphate buffered saline (PBS) for 15 min, then the cells were treated with 2 mg/mL glycine and 0.5% Triton X-100. To detect proliferating cells, specimens were incubated with 100 μL EdU Click-iT mixture (1 mL EdU Click-iT mixture contains 860 μL 1 × Click iT EdU reaction buffer, 40 μL CuSO_4_, 3 μL kFluor488-azide, and 100 μL reaction buffer additive) for 30 min at room temperature in the dark. Cells were incubated with 4’,6-diamidino-2-phenylindole (DAPI) for 5 min. Images were acquired using an inverted fluorescence microscope (Leica DMIL LED, Leica Microsystems CMS GmbH, Wetzar, Germany). Cell proliferation was determined by calculating the percentage of EdU-stained cells among the total cells.

### Cell Migration Assay

Cell migration was evaluated using a scratch test. C2C12 myoblasts were seeded in six-well plates, transfected with siRNA, and incubated for 48 h until they formed a confluent layer. Then, a scratch was generated in each well using a pipette tip, after which the cells were washed with PBS. The cells were then incubated in serum-free medium at 37°C under 5% CO_2_, and images were acquired at 0, 6, 12, and 24 h after scratch formation. Image J software was used to standardize the results.

### Immunofluorescence Staining

Cells were fixed in 4% paraformaldehyde for 15 min, permeabilized using 0.5% Triton X-100 in PBS, and blocked with 5% goat serum (Beyotime Institute of Biotechnology). The cells were then incubated with primary anti-MyHC antibody (1:80) overnight at 4°C. Cell nuclei were stained using DAPI (Beyotime Institute of Biotechnology) for 5 min at room temperature. Cells were then washed for 5 min in PBS three times. Images were acquired using an inverted fluorescence microscope (Leica DMIL LED, Leica Microsystems CMS GmbH). Fusion indices were calculated as the percentage of nuclei with two or more nuclei in the fused myotubes out of the total nuclei.

### High-Throughput Sequencing

The RNA sequencing results of differentiating myoblasts were analyzed by Aksomics Inc. (Shanghai, China). Gene ontology (GO) and Kyoto Encyclopedia of Genes and Genomes (KEGG) pathway analyses were performed using standard computational enrichment methods. Gene set enrichment analysis (GSEA) was performed using the Molecular Signatures Database^1^. The data were deposited in the Gene Expression Omnibus under accession number GSE164407.

### Statistical Analyses

Data are reported as the mean ± standard deviation. The statistical significance between two groups was assessed using two-tailed Student’s *t*-tests. Comparisons among three or more groups were performed using one-way analysis of variance; when significant differences were found, the least significant difference was calculated. *P* < 0.05 was taken to indicate statistical significance. All data were analyzed using SPSS 21.0 (IBM Corporation, Armonk, NY, United States).

## Results

### CNN3 Was Upregulated During Myogenic Differentiation and Muscle Regeneration

**FIGURE 1:**
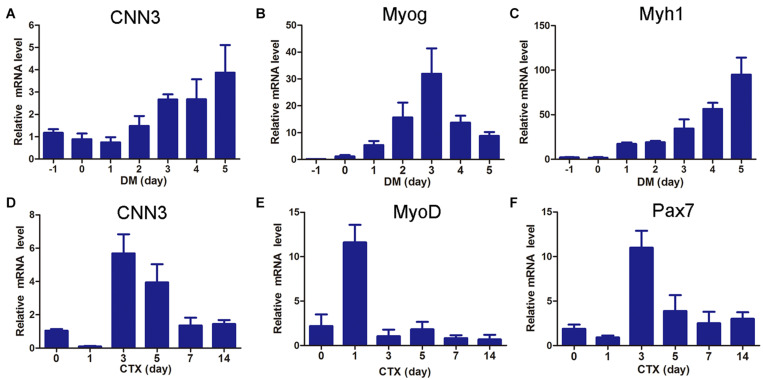
CNN3 was upregulated during myogenesis and muscle regeneration. **(A–C)** qRT-PCR results showing the expression patterns of **(A)** CNN3, **(B)** Myog, and **(C)** Myh1 during myoblast differentiation. **(D–F)** Hindlimb muscles were injected with cardiotoxin (CTX) and Tibialis anterior (TA) muscles were harvested at 0, 1, 3, 5, 7, and 14 days post-injury for **(D)** CNN3, **(E)** MyoD, and **(F)** Pax7 mRNA analyses.

To investigate the role of CNN3 in muscle cell differentiation, we examined the expression of CNN3, Myog, and Myh1 in differentiating myoblasts. The levels of CNN3 and Myh1 gradually increased during differentiation of C2C12 cells, whereas that of Myog increased during the early stages of differentiation but then decreased following myotube maturation (Figures 1A–C). To examine CNN3 expression during muscle regeneration, we injected CTX into the hindlimb muscles to induce muscle injury and regeneration (Singh et al., 2014; Wang et al., 2019). The expression levels of CNN3, as well as Pax7 and MyoD, were significantly increased during the early stages of regeneration but then decreased once the newly formed fibers had reached maturation and regeneration was complete (Figures 1D–F).

### Effects of CNN3 on Proliferating Myoblasts

**FIGURE 2:**
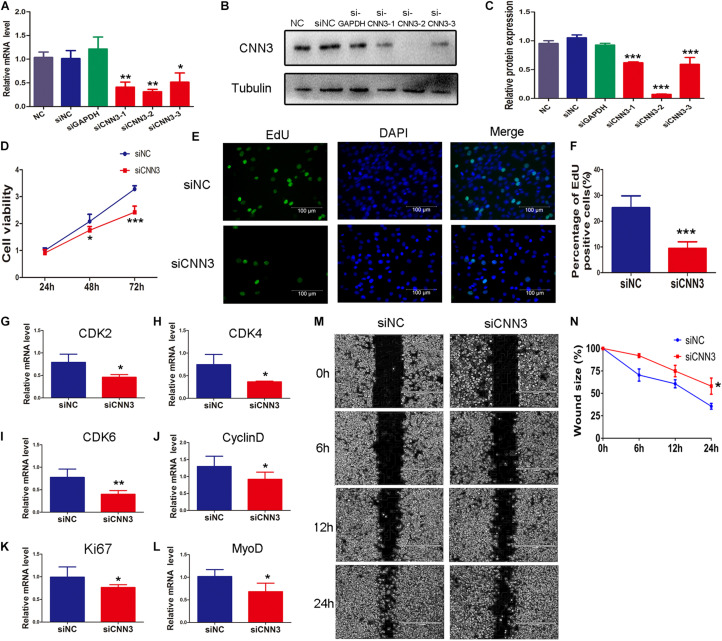
The effects of CNN3 knockdown on proliferating myoblasts. **(A)** The relative mRNA expression of CNN3 after RNAi-mediated knockdown. **(B,C)** Western blot analysis of CNN3 and tubulin after CNN3 knockdown. **(D)** Cell viability assessed using the CCK8 assay. **(E)** Cell proliferation quantified by EdU labeling. The green color indicates EdU labeling, and the blue color indicates DAPI nuclear staining. **(F)** The percentage of EdU-positive cells among the total number of cells detected by DAPI nuclear staining. **(G–L)** Downregulated expression of **(G)** CDK2, **(H)** CDK4, **(I)** CDK6, **(J)** cyclin D, **(K)** Ki67, and **(L)** MyoD in the CNN3 knockdown group. **(M)** Phase contrast microscopy images of migrating C2C12 cells at 0, 6, 12, and 24 h after scratching. **(N)** Quantitative analysis of cells the percentage of migrating into the scratch area at 0, 6, 12, and 24 h after scratching. *P < 0.05, **P < 0.01, ***P < 0.001 compared with the NC siRNA group.

To explore the function of CNN3 in proliferating myoblasts, we examined C2C12 cell proliferation following siRNA knockdown of CNN3. First, we selected an siRNA that inhibited CNN3 expression by ∼70% (Figures 2A–C). We then transfected C2C12 cells with CNN3 and negative control (NC) siRNA. The CCK8 analysis indicated that CNN3 knockdown reduced C2C12 myoblast proliferation compared with the NC siRNA group at 48 h and 72 h (Figure 2D). We then performed EdU labeling to evaluate myoblast proliferation. Our results showed that CNN3 knockdown decreased the proportion of EdU-positive cells compared with the NC siRNA group (Figures 2E,F). Cyclin-dependent kinase (CDK)-2, -4, -6, and cyclin D are essential for the cell cycle (Lim and Kaldis, 2013). Ki67 is a marker of cellular proliferation (Sobecki et al., 2017), and MyoD is a transcriptional activator that regulates muscle regeneration (Cichewicz et al., 2018). Consistent with the CCK8 and EdU assay results, qRT-PCR analysis showed that CNN3 knockdown strongly downregulated the expression of CDK-2, -4, -6, cyclin D, Ki67, and MyoD (Figures 2G–L) compared with the NC siRNA group, which suggests that knockdown of CNN3 may suppress C2C12 myoblast proliferation.

We then applied a scratch wound-healing assay to investigate the role of CNN3 in C2C12 myoblast migration and proliferation. CNN3 knockdown caused a ∼25% reduction in cell migration compared with the NC siRNA group. These results suggest that CNN3 likely has an important function in C2C12 cell migration and proliferation (Figures 2M,N).

### Effects of CNN3 on Differentiating Myoblasts

**FIGURE 3:**
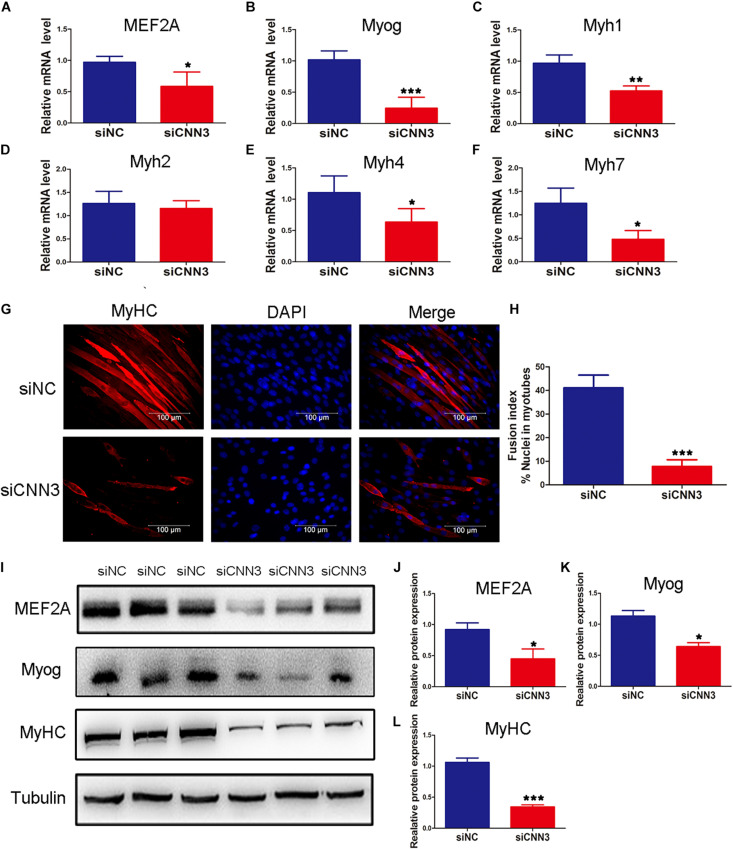
Effects of CNN3 on differentiating myoblasts. **(A–F)** The relative mRNA expression of **(A)** MEF2A, **(B)** Myog, **(C)** Myh1, **(D)** Myh2, **(E)** Myh4, and **(F)** Myh7 between the NC siRNA (siNC) and CNN3 knockdown (siCNN3) groups. **(G)** Representative images of differentiating C2C12 cells stained with anti-MyHC antibodies. The red color indicates MyHC staining, and the blue color indicates DAPI nuclear staining. Scale bars = 100 μm. **(H)** The fusion indices quantified after CNN3 knockdown. **(I)** Western blot analysis of MEF2A, Myog, and MyHC. **(J–L)** Quantification of the Western blot bands for **(J)** MEF2A, **(K)** Myog, and **(L)** MyHC by Image J, using tubulin as the internal control. *P < 0.05, **P < 0.01, ***P < 0.001 compared with the siNC group.

To examine the function of CNN3 in differentiating myoblasts, we measured the expression of differentiation markers at several time points following transfection of CNN3 siRNA. Compared with the NC siRNA group, the mRNA levels of MEF2A, Myog, Myh1, Myh4, and Myh7 were downregulated in CNN3 knockdown cells (Figures 3A–F). Myh1, Myh2, Myh4, and Myh7 are associated with different types of muscle fibers. Myh7-positive type I fibers are slim and long, whereas Myh1-, Myh2-, and Myh4-positive type II fibers are thick and short (Chen et al., 2019). Myh1, Myh4, and Myh7 expression levels were markedly decreased following CNN3 siRNA transfection. The changes in Myh1, Myh4, and Myh7 expression suggest that CNN3 plays a role in the differentiation of MyHC isoforms. The MyHC immunofluorescence staining results showed the knockdown of CNN3 inhibited C2C12 cell differentiation (Figure 3G) and decreased fusion index (Figure 3H). Western blot analysis in differentiated C2C12 myoblasts indicated that CNN3 knockdown resulted in significantly lower levels of differentiation marker proteins, such as MEF2A, Myog, and MyHC (Figures 3I–L).

### CNN3 Knockdown Inhibited Protein Synthesis Associated With AKT/mTOR and AMPK/mTOR Signaling Pathways

**FIGURE 4:**
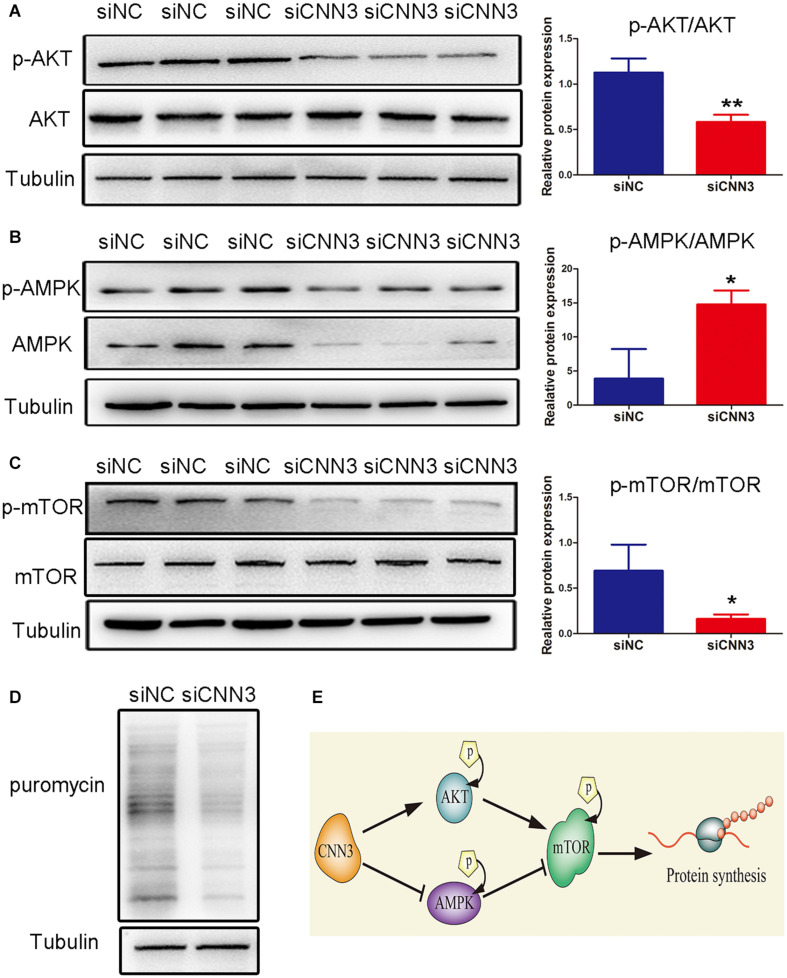
CNN3 knockdown suppressed cell protein synthesis in C2C12 cells via the AKT/mTOR and AMPK/mTOR pathways. **(A–C)** Western blot analysis of the protein levels of **(A)** p-AKT and AKT, **(B)** p-AMPK and AMPK, and **(C)** p-mTOR and mTOR in the NC siRNA (siNC) and CNN3 knockdown (siCNN3) groups. The Western blot bands were quantified using Image J, and tubulin was used as the internal control. **(D)** Newly synthesized proteins detected by Western blot analysis using an anti-puromycin antibody in the siNC and siCNN3 groups.**(E)** A schematic representation of our proposed mechanism for the role of CNN3 in protein synthesis in C2C12 cells.*P < 0.05, **P < 0.01 compared with the siNC group.

Next, we analyzed the protein levels of p-AKT, AKT, p-AMPK, AMPK, p-mTOR, and mTOR. We found that the levels of p-AMPK/AMPK were increased, whereas those of p-AKT/AKT and p-mTOR/mTOR were decreased, in CNN3 knockdown cells compared with NC siRNA group cells (Figures 4A–C). Because mTOR is a positive regulator of protein synthesis, we labeled C2C12 cells with puromycin to determine whether CNN3 knockdown suppressed protein synthesis. Our results indicated that the rate of protein synthesis was dramatically lower in the CNN3 siRNA group than in the NC siRNA group (Figure 4D). Our results suggest that CNN3 knockdown decreased protein synthesis, and that this effect may involve the AKT/mTOR and AMPK/mTOR signaling pathways. And the proposed mechanism of CNN3 in protein synthesis of C2C12 cells is shown in Figure 4E.

### Restoration of the mTOR Pathway Decreased the Inhibitory Effects of CNN3 Knockdown on C2C12 Cell Proliferation, Differentiation, and Protein Synthesis

**FIGURE 5:**
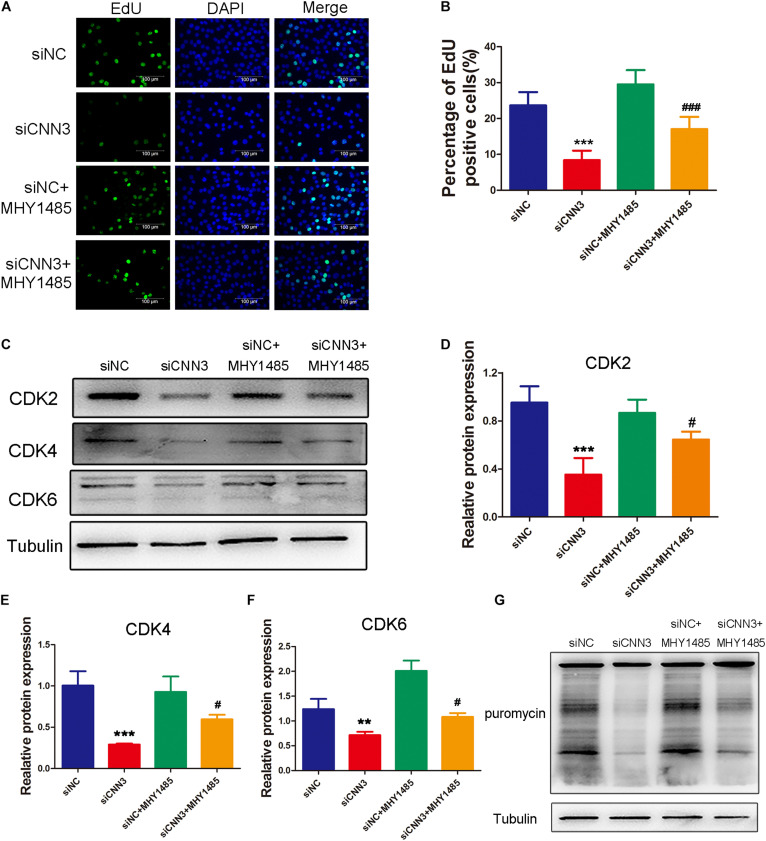
MHY1485 treatment restored myoblast proliferation and protein synthesis in CNN3 knockdown cells. **(A)** Fluorescence images showing EdU-labeled cells to assess cell proliferation. The green color indicates EdU labeling, and the blue color indicates DAPI nuclear staining. **(B)** The percentage of EdU-positive cells among the total number of cells detected by DAPI nuclear staining. **(C)** Western blot analysis of CDK2, CDK4, and CDK6. **(D–F)** Quantification of the Western blot bands for **(D)** CDK2, **(E)** CDK4, and **(F)** CDK6 by Image J, using tubulin was used as the internal control. **(G)** Newly synthesized proteins detected by Western blot analysis using an anti-puromycin antibody. **P < 0.01, ***P < 0.001 compared with the NC siRNA (siNC) group; ^#^*P* < 0.05, ^###^*P* < 0.001 compared with the CNN3 knockdown (siCNN3) group.

To determine whether the inhibited proliferation induced by CNN3 knockdown involves the mTOR pathway, we treated CNN3 knockdown cells with the mTOR activator MHY1485. In proliferating cells, EdU labeling and Western blot analyses revealed that the inhibitory effect of CNN3 knockdown on cell proliferation was reversed by MHY1485 treatment (Figures 5A,B). Furthermore, MHY1485 treatment led to increased expression of CDK-2, -4, and -6 (Figures 5C–F). The puromycin labeling assay indicated that MHY1485 partially restored the reduction in protein synthesis induced by CNN3 knockdown (Figure 5G).

**FIGURE 6:**
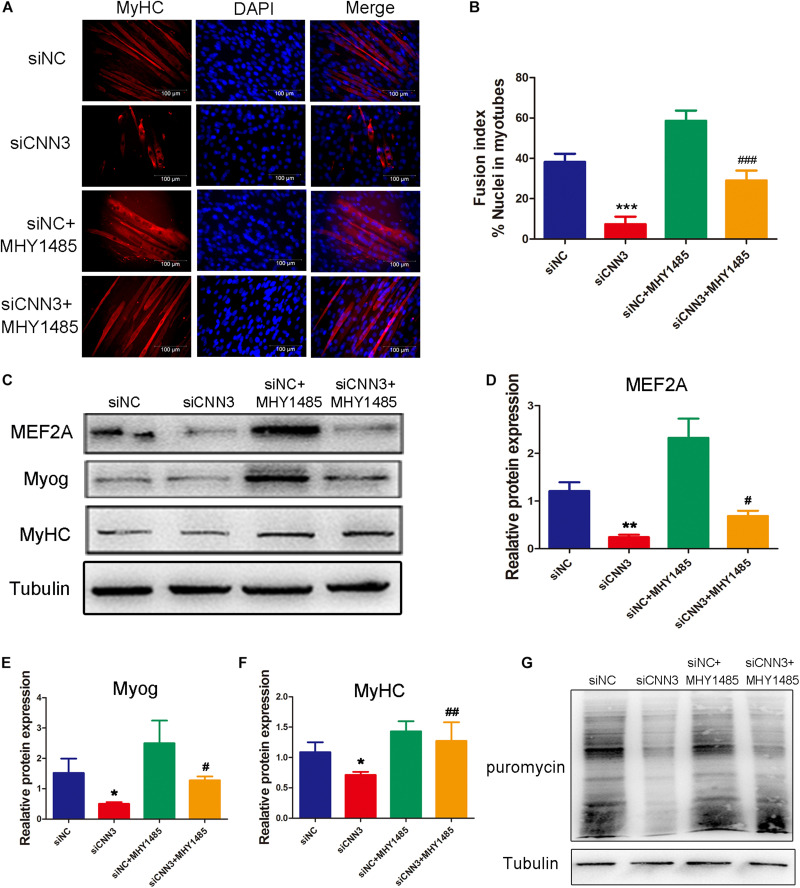
MHY1485 treatment restored myogenic differentiation and protein synthesis in CNN3 knockdown cells. **(A)** Representative images of differentiating C2C12 cells stained with an anti-MyHC antibody. The red color indicates MyHC staining, and the blue color indicates DAPI nuclear staining. Scale bars = 100 μm. **(B)** The fusion indices quantified for each group. **(C)** Western blot analysis of CDK2, CDK4, and CDK6. **(D–F)** Quantification of the Western blot bands by Image J, using tubulin as the internal control. **(G)** Newly synthesized proteins detected by Western blot analysis using an anti-puromycin antibody. *P < 0.05, **P < 0.01, ***P < 0.001 compared with the NC siRNA (siNC) group; ^#^*P* < 0.05, ^##^*P* < 0.01, ^###^*P* < 0.001 compared with the CNN3 knockdown (siCNN3) group.

At the differentiation stage, the percentage of MyHC-positive cells (Figure 6A), fusion index (Figure 6B), and levels of differentiation markers (MEF2A, Myog, and MyHC; Figures 6C–F) were significantly higher after treatment with MHY1485 compared with untreated CNN3 knockdown cells. Western blot analysis revealed that protein synthesis was restored in the MHY1485-treated CNN3 knockdown group (Figure 6G).

Our results indicated that restoring the mTOR pathway decreased the inhibitory effect of CNN3 knockdown on C2C12 cell proliferation, differentiation, and protein synthesis.

### GO and KEGG Pathway Analyses Revealed Potential CNN3-Regulated Mechanisms

**FIGURE 7:**
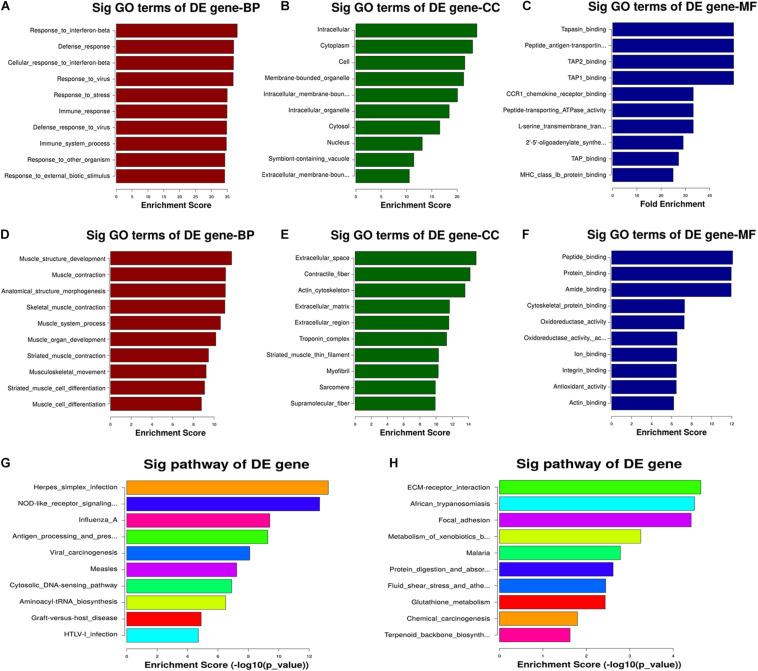
GO and KEGG pathway analyses revealed the potential mechanism regulated by CNN3. **(A–F)** Gene ontology (GO) terms associated with the **(A–C)** upregulated and **(D–F)** downregulated mRNAs after CNN3 knockdown. The top 10 GO terms with the highest enrichment scores for the biological process (BP), cellular component (CC), and molecular function (MF) categories in the NC siRNA (siNC) and CNN3 knockdown (siCNN3) cells. **(G,H)** The KEGG pathways with the 10 highest enrichment scores for the **(G)** upregulated and **(H)** downregulated transcripts.

The GO analysis revealed that the transcripts downregulated in the CNN3 knockdown group relative to the NC siRNA group are primarily involved in biological processes including muscle structure development, muscle contraction, muscle system processes, muscle organ development, striated muscle contraction, musculoskeletal movement, and muscle cell differentiation (Figures 7A–F). We then performed KEGG pathway analysis to identify the pathways and molecular interactions associated with the differentially expressed transcripts. The herpes simplex infection, nucleotide-binding oligomerization domain (NOD)-like receptor signaling, and influenza pathways were the top pathways associated with the upregulated mRNAs. The top three enriched KEGG pathways associated with the downregulated mRNAs were extracellular matrix receptor interaction, African trypanosomiasis, and focal adhesion (Figures 7G,H).

### GSEA Showed Enriched Among Differentially Regulated Genes in CNN3 Knockdown C2C12 Cells

**FIGURE 8:**
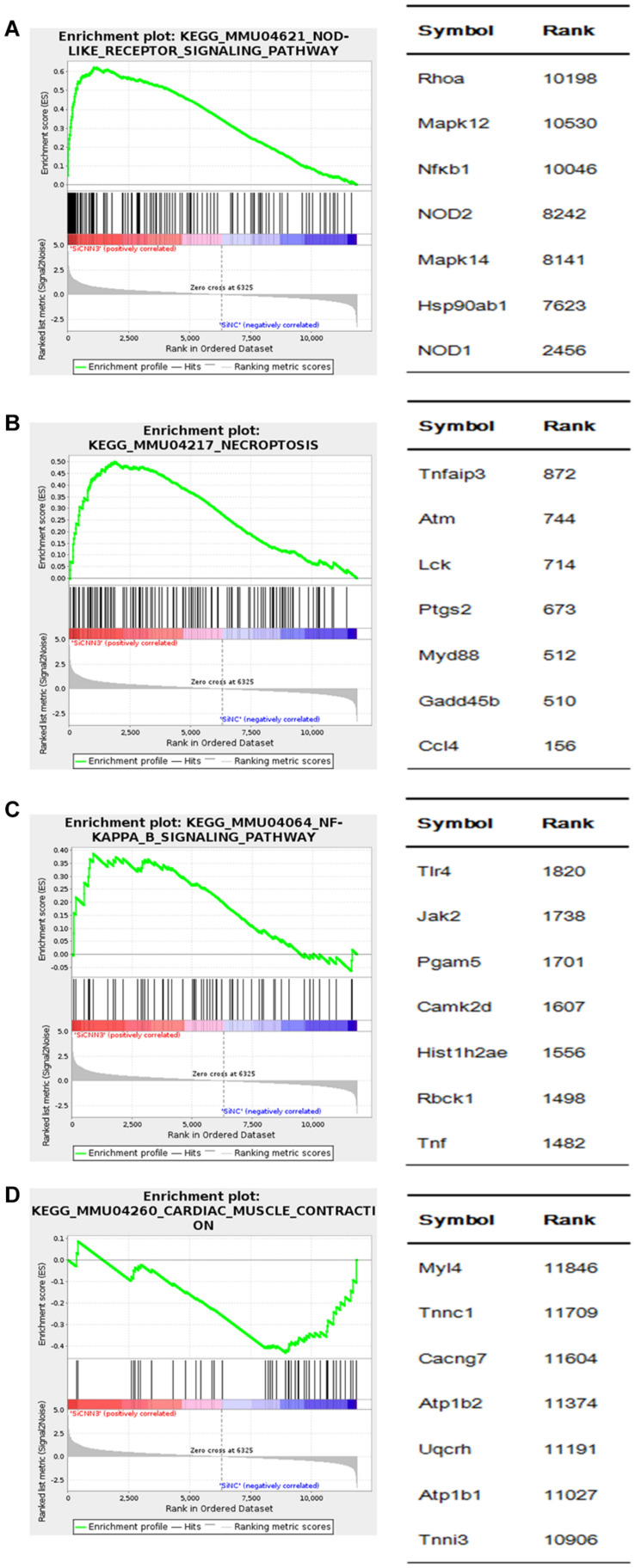
GSEA of differentially expressed genes in CNN3 knockdown C2C12 cells. GSEA results are shown. The upregulated genes in **(A)** nucleotide-binding oligomerization domain (NOD)-like receptor signaling pathway [normalized enrichment score (NES) = 1.869; *P* = 0.000], **(B)** necroptosis signaling pathway (NES = 1.698; *P* = 0.000), **(C)** NF-κB signaling pathway (NES = 1.428; *P* = 0.000), and the downregulated genes in **(D)** cardiac muscle contraction signaling pathway (NES = -1.349; *P* = 0.000).

We then performed GSEA to further analyze the gene sets that were differentially regulated in CNN3 knockdown cells. The upregulated genes in CNN3 knockdown group are enriched in pathways included the NOD-like receptor signaling pathway [normalized enrichment score (NES) = 1.869, *P* = 0.000], necroptosis signaling pathway (NES = 1.698, *P* = 0.000), and NF-κB signaling pathway (NES = 1.428, *P* = 0.000) (Figures 8A–C). The downregulated genes in CNN3 knockdown group are enriched in pathways included cardiac muscle contraction signaling pathway (NES = 1.349; *P* = 0.000) (Figure 8D).

## Discussion

Myogenesis is a complex process that consists of several major phases, including cell proliferation, differentiation, and fusion into myofibers (Horak et al., 2016). Although the mechanisms of myogenesis have been investigated extensively, the processes mediated by CNN3 remain largely unknown.

In this study, we investigated the effects of CNN3 on C2C12 myoblast proliferation, migration, differentiation, fusion, and protein synthesis. We found that CNN3 expression gradually increased during differentiation in C2C12 cells, and that during injury, the Pax7 and MyoD genes were upregulated. Our model of CTX-induced injury in skeletal muscle revealed that the general trend of CNN3 gene expression was similar to that of Pax7 and MyoD, suggesting that CNN3 may be involved in myogenesis.

Proliferation and migration of myoblasts are essential for muscle development, as they facilitate cellular alignment in preparation for differentiation and fusion (Wang et al., 2018). CNN3 plays a pivotal role in promoting the migration of cancer cells, skin cells, and trophoblast invasion (Shibukawa et al., 2010; Daimon et al., 2013; Kim et al., 2018; Nair et al., 2019; Xia et al., 2020). In xenografted cervical cancer cells, CNN3 acted as an oncogene, promoting cell viability and motility *in vitro* by altering the expression of ribosomal protein lateral stalk subunit P1 (Xia et al., 2020). CNN3 can upregulate multiple oncogenic pathways, such as those involving ERK1/2, β-catenin, c-Jun, heat shock protein 60, and mutant p53 (Nair et al., 2019). CNN3 knockdown decreased cell motility and contractility in primary fibroblasts, which are involved in actin stress fiber remodeling (Daimon et al., 2013). On the other hand, CNN3 knockdown in neuronal stem cells promoted proliferation, resulting in a higher number of stem cells within each sphere formed (Junghans and Herzog, 2018). Based on our results, we propose that CNN3 knockdown may suppress C2C12 myoblast proliferation and migration.

Myoblast differentiation and fusion are necessary for muscle formation during development and regeneration (Quinn et al., 2017). CNN3 is a negative regulator of trophoblast fusion via suppression of actin cytoskeletal rearrangement and syncytium formation (Shibukawa et al., 2010). Shibukawa et al. (2013) suggested that CNN3 impairs cellular fusion, and that it regulates actin cytoskeletal rearrangement via phosphorylation of the CNN3-specific C-terminal region. In that study, CNN3 knockdown revealed that CNN3 promotes skeletal myosin expression and fusion. However, in our research, we found that the expression of myogenic differentiation factors was delayed, and that the fusion index was decreased by CNN3 knockdown under myogenic differentiation conditions. These discrepancies may be caused by different cultural conditions.

Taken together, we propose that CNN3 expression regulates muscle development; nevertheless, the precise functions of CNN3 in muscle development are poorly understood. mTOR regulates proliferation and differentiation in various cell lines, which is associated with protein synthesis (Morita et al., 2015). Our results revealed that CNN3 promoted protein synthesis in C2C12 cells at both the proliferation and differentiation stages. mTOR is likely regulated by AKT and AMPK. AKT is activated during skeletal muscle hypertrophy, while AMPK activation induces muscle atrophy (Scrimgeour et al., 2001; Chen et al., 2017). Here, we speculate that the AKT and AMPK pathways participate in CNN3-regulated myogenesis. Our Western blot analyses confirmed the induction of AKT/mTOR and AMPK/mTOR by CNN3.

Multiple pathways contribute to skeletal muscle development. Tang et al. (2014) performed paired microRNA and mRNA profiling in the prenatal skeletal muscle of pigs and revealed that miR-1 regulated skeletal muscle development by targeting CNN3. In this study, we performed GO and KEGG analyses, and GSEA to explore the potential role of CNN3 in muscle development. We found that the genes upregulated by CNN3 knockdown were involved in necroptosis, apoptosis, and inflammation signaling pathways, while the downregulated genes were associated with muscle structure development, muscle contraction, striated muscle contraction, and muscle cell differentiation. GO analysis revealed that the differentially expressed mRNAs in siNC and siCNN3 groups were involved in actin-cytoskeleton, endoplasmic reticulum (ER) transport, MHC class Ib protein binding, etc. CNN3 was reported to take part in actin-cytoskeleton based activities in myoblast fusion and embryonic brain and systemic development (Shibukawa et al., 2013; Junghans and Herzog, 2018). Rock-dependent CNN3 regulates actin cytoskeletal rearrangement via phosphorylation of the CNN3-specific C-terminal region, which impairs the fusion of myoblasts (Shibukawa et al., 2013). Knockout of CNN3 in mice lead to massive malformations in embryonic and neonatal lethality by regulating actin cytoskeletal reorganization (Junghans and Herzog, 2018). Notably, the results of our KEGG and GSEA indicated that the NOD-like receptor (NLR) signaling pathway was affected by CNN3 knockdown. The NLR signaling pathway is over-active in patients with muscular dystrophy. Furthermore, lipopolysaccharides directly induce muscular atrophy by engaging the Toll-like receptor 4 and/or NOD2 signaling pathways in muscle cells, which then suppress AKT/mTOR/FOXO1 signaling (Liu et al., 2016). Sustained NLRP3 inflammasome activation in dystrophin-deficient mdx mice led to muscle fiber loss and increased inflammation (Chang et al., 2019). In Duchenne muscular dystrophy patients, the NLRP3 inflammasome is upregulated, and ablation of NLRP3 attenuated the dystrophic phenotype (Boursereau et al., 2018). These analyses indicate that inhibition of CNN3 may activate the NLR signaling pathway, thus causing myocyte atrophy.

One limitation to our study is that we did not overexpress CNN3; however, this was because of the naturally high CNN3 expression in C2C12 cells. Our GSEA provided insight into the potential molecular mechanisms governed by CNN3 during skeletal muscle development. Further studies are needed to deepen our understanding of the involvement of CNN3 in various pathways such as NLR. Besides, since the isoforms of CNN have largely conserved structures and functions, we also detected the expression of CNN2 in differentiating myoblasts with and without siCNN3 treatment. As the result showed, CNN2 had a slight increase in mRNA expression, but there was no significant difference in protein expression between two groups (Supplementary Figure 2). We speculated CNN3 in C2C12 differentiation do not work through the known conservative functional domains shared by CNN2. Other regulating mechanisms processing differentiation of CNN3 in C2C12 merits further investigation.

## Conclusion

In this study, we knocked down CNN3 in C2C12 cells to explore its functions. CNN3 knockdown caused reductions in cell proliferation, migration, myogenic differentiation, fusion, and protein synthesis via the AKT/mTOR and AMPK/mTOR pathways; these effects were reversed by MHY1485 treatment. The KEGG and GSEA indicated that inhibiting CNN3 may activate several pathways, including the NOD-like receptor pathway and pathways involved in necroptosis, apoptosis, and inflammation.

## Data Availability Statement

The datasets presented in this study can be found in online repositories. The names of the repository/repositories and accession number(s) can be found below: https://www.ncbi.nlm.nih.gov/, GSE164407.

## Ethics Statement

The animal study was reviewed and approved by the Ethics Committee of Guangdong Second Provincial General Hospital.

## Author Contributions

RC conceived and designed the experiments. YS, SL, and SZ performed the *in vitro* experiments. SL and HS performed the *in vivo* experiments. CL and TJ analyzed the data. YS, CL, and RC wrote the manuscript. All authors read and approved the final version.

## Conflict of Interest

The authors declare that the research was conducted in the absence of any commercial or financial relationships that could be construed as a potential conflict of interest.

## Footnotes

1 http://software.broadinstitute.org/gsea/msigdb
