# Supplementary material for: Knockdown of CNN3 Impairs Myoblast Proliferation, Differentiation, and Protein Synthesis via the mTOR Pathway
Source: Front Physiol. 2021 Jul 8;12:659272. doi: 10.3389/fphys.2021.659272 (PMC8295729; doi:10.3389/fphys.2021.659272)
Supplement: Supplementary file 1 [file Data_Sheet_1.docx]

**Supplementary**

**Title:** **Knockdown of CNN3 impairs myoblast proliferation, differentiation and protein synthesis through** **mTOR pathway**

**Authors:**

Yanling She^1*^, Cheng Li^1*^, Ting Jiang^2^, Si Lei^1^, Shanyao Zhou^1^, Huacai Shi^1^ and Rui Chen^1^

**Address of the institution:**

^1^Guangdong Traditional Medical and Sports Injury Rehabilitation Research Institute, Guangdong Second Provincial General Hospital, Guangzhou, Guangdong 510317, China.

^2^Department of Radiology, The Third Affiliated Hospital, Sun Yat-sen University, 600 Tian He Road, Guangzhou 510630, China

^*^ Contributed equally

**Correspondence should be addressed to** Rui Chen; rui.c.med@163.com

**Corresponding author at:** Guangdong Traditional Medical and Sports Injury Rehabilitation Research Institute, Guangdong Second Provincial General Hospital, 466 Xin Gang Zhong Road, Guangzhou 510317, China.

**Telephone number:** +86-20-89168760

**Suppl. Table 1.** siRNA sequences

| siRNA | sence (5′ to 3′) | anti-sence (5′ to 3′) |
| --- | --- | --- |
| NC siRNA | UUCUCCGAACGUGUCACGUTT | ACGUGACACGUUCGGAGAATT |
| CNN3 siRNA1 | GCCUAGGCAUUGGCACCAATT | UUGGUGCCAAUGCCUAGGCTT |
| CNN3 siRNA2 | CCAUUGACAUUGGCGUUAATT | UUAACGCCAAUGUCAAUGGT |
| CNN3 siRNA3 | GGGCACCAAUGGGUCGGAATT | UUCCGACCCAUUGGUGCCCTT |
| GAPDH siRNA | CACUCAAGAUUGUCAGCAATT | UUGCUGACAAUCUUGAGUGAG |

**Suppl. Table 2.**  Primers for the detected mRNAs

| mRNA | Forward primer (5′ to 3′) | Reverse primer (5′ to 3′) |
| --- | --- | --- |
| 18S | GTAACCCGTTGAACCCCATT | CCATCCAATCGGTAGTAGCG |
| CNN3 | TTCAGGGATGGATCTCAAGG | AGCCATCTCTCCAGTCCTCA |
| Myog | GGCAATGCACTGGAGTTCG | AGCCGCGAGCAAATGATC |
| Myh1 | CTCTTCCCGCTTTGGTAAGTT | CAGGAGCATTTCGATTAGATCCG |
| Myh2 | AAGTGACTGTGAAAACAGAAGCA | GCAGCCATTTGTAAGGGTTGAC |
| Myh4 | TTGAAAAGACGAAGCAGCGAC | AGAGAGCGGGACTCCTTCTG |
| Myh7 | ACTGTCAACACTAAGAGGGTCA | TTGGATGATTTGATCTTCCAGGG |
| MEF2A | CAGGTGGTGGCAGTCTTGG | TGCTTATCCTTTGGGCATTCAA |
| CDK2 | GCGACCTCCTCCCAATATCG | GTCTGATCTCTTTCCCCAACTCT |
| CDK4 | ATGGCTGCCACTCGATATGAA | TCCTCCATTAGGAACTCTCACAC |
| CDK6 | GGCGTACCCACAGAAACCATA | AGGTAAGGGCCATCTGAAAACT |
| Ki67 | ATCATTGACCGCTCCTTTAGGT | GCTCGCCTTGATGGTTCCT |
| cyclin D | GCGTACCCTGACACCAATCTC | CTCCTCTTCGCACTTCTGCTC |
| MyoD | CCACTCCGGGACATAGACTTG | AAAAGCGCAGGTCTGGTGAG |


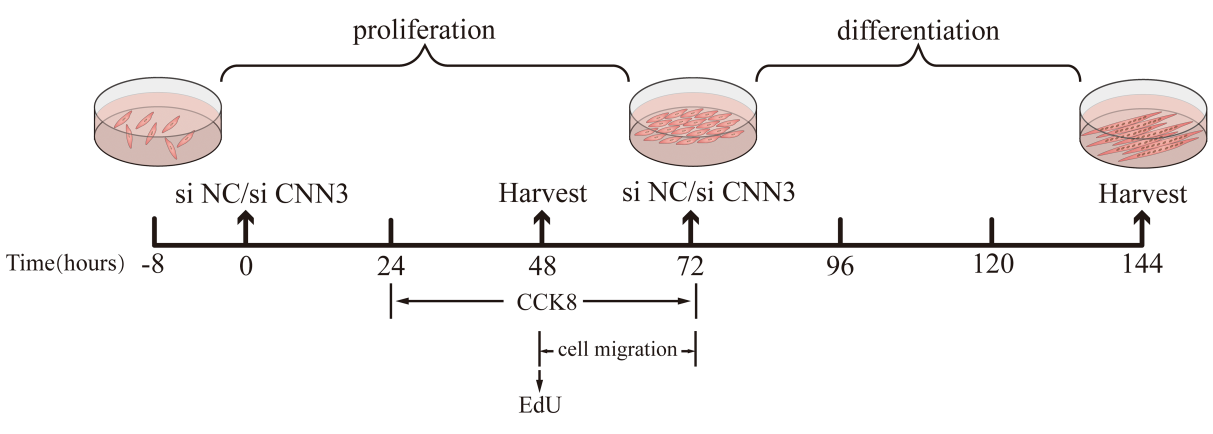


**Suppl. Figure 1.** Timeline of the experiments


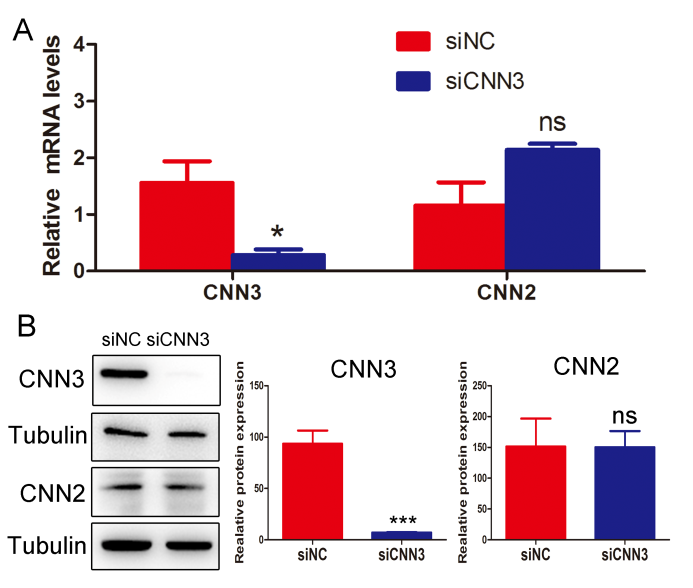


**Suppl. Figure 2.** The relative expression of CNN2 in siNC and siCNN3 groups during C2C12 differertaration

(A) The relative mRNA expression of CNN3 and CNN2 in siNC and siCNN3 groups. (B) Western blot analysis of CNN3 and CNN2 in siNC and siCNN3 groups. **P*< 0.05, ****P*< 0.001 compared with the siNC group, ns: not significant.
